# Integrating metabolomic and transcriptomic analyses to identify candidate genes and transcriptional regulators associated with triterpenoid saponin accumulation in developing *Momordica charantia* fruits

**DOI:** 10.3389/fpls.2026.1868077

**Published:** 2026-10-06

**Authors:** Junmiao Fan, Dongmei Lian, Yunfa Yao, Zhou Li, Lidong Wu, Shaoping Zhang, Bizhen Lin, Zhengfeng Lai

**Affiliations:** 1 Institute of Subtropical Agriculture, Fujian Academy of Agricultural Sciences, Zhangzhou, China; 2 Sanming Academy of Agricultural Sciences, Sanming, China

**Keywords:** biosynthetic pathway, CYP450, fruit, metabolomics, *Momordica charantia*, transcriptomics, triterpenoid saponin, UPLC-MS/MS

## Abstract

**Background:**

*Momordica charantia* is an economically and pharmacologically important vegetable, with its medicinal value largely attributed to its diverse triterpenoid saponins. Although the decline of total saponin content during fruit development is well-documented, the precise metabolic profiles and the underlying molecular regulatory mechanisms governing individual saponin accumulation remain poorly understood.

**Methods:**

We performed integrated metabolomic and transcriptomic analyses on fruits of the *Momordica charantia* cultivar ‘Ruyu No. 45’ (‘RY45’) at 10 and 15 days post-anthesis (DPA). Metabolites were profiled using ultra-performance liquid chromatography-tandem mass spectrometry, and transcriptomes were analyzed via RNA sequencing. Weighted gene co-expression network analysis and Pearson correlation analysis were applied to identify transcription factors and structural genes (cytochrome P450 monooxygenases and UDP-glycosyltransferases) potentially involved in saponin biosynthesis.

**Results:**

We identified 473 terpenoid metabolites, of which 56 triterpenoid saponins were tentatively identified (Metabolomics Standards Initiative Level 2), comprising cucurbitane-type (44.6%), oleanane-type (53.6%), and ursane-type (1.8%). Total saponin content decreased significantly from 10 DPA to 15 DPA, with 27 individual triterpenoid saponins showing significant downregulation. Transcriptome analysis revealed 3,291 differentially expressed genes, including 50 candidate genes potentially involved in triterpenoid saponin biosynthesis. Candidate structural genes (*McCYP72A219*, *McCYP86A7*, *McCYP704C1*, *McUGT73C6*, *McUGT84B1* and *McβAS*) were markedly downregulated at 15 DPA and positively correlated with multiple saponin contents. Additionally, transcription factors *McbHLH93–1* and *McNAC90–1* were identified as co-expressed with candidate structural genes. Transient overexpression of *McCYP72A219* was associated with increased total saponin content, providing preliminary functional clues for its potential role in saponin accumulation.

**Conclusion:**

This study provides a systematic overview of the metabolic decline of triterpenoid saponins in bitter gourd at 15 DPA compared to 10 DPA, identifies a suite of candidate structural genes and transcription factors and constructs a preliminary co-expression regulatory network as an exploratory analysis. These findings offer new insights into the transcriptional regulation of triterpenoid saponin accumulation in bitter gourd and lay a molecular foundation for future metabolic engineering and quality improvement. However, the proposed regulatory relationships are primarily correlative and require further genetic and biochemical validation.

## Introduction

*Momordica charantia* (bitter gourd) is a widely cultivated vegetable renowned not only for its culinary versatility but also for its significant pharmacological value. The pharmacological efficacy of bitter gourd has been extensively validated, demonstrating significant potential in hypoglycemic regulation (Jia et al., 2017), the management of diabetes and obesity (Fan et al., 2019), anti-tumor activity (Dandawate et al., 2016), and the treatment of hepatic injuries (Tian et al., 2024). Triterpenoid saponins are essential bioactive constituents of bitter gourd fruit. These compounds are predominantly classified into two distinct triterpene skeleton types: cucurbitane-type triterpene glycosides (CTGs) and oleanane-type triterpene glycosides (OTGs) (Nakamura et al., 2006; Tan et al., 2008). Notably, CTGs, a class of tetracyclic triterpenoids characteristic of the *Cucurbitaceae* family, are recognized as the primary contributors to the fruit’s distinct bitter taste as well as its anti-diabetic and hypoglycemic activities (Harinantenaina et al., 2006; Keller et al., 2011). Meanwhile, OTGs are widely distributed across various plant species, and their potential anti-diabetic properties have been extensively documented (Ayeleso et al., 2017).

Since the beginning of the 21st century, various researchers have identified 98 triterpenoid saponins from bitter gourd using spectroscopic analyses (Murakami et al., 2001; Akihisa et al., 2007; Hsiao et al., 2013; Liaw et al., 2015). The majority of these compounds belong to the cucurbitane-type skeleton, while only a minor fraction are of the oleanane-type. Among them, four CTGs (momordicoside K, momordicoside L, momordicine I, and momordicine II) were found to be bitter (Lee et al., 2021), while momordicoside M, momordicoside K, momordicoside Q, momordicine I, and momordicine II have been reported to possess antidiabetic activities (Tan et al., 2008; Hsiao et al., 2013; Sun et al., 2021). Furthermore, the composition of these triterpenoid saponins can be significantly influenced by variations in plant organs, cultivars, or growth habitats (Zhou et al., 2019). Previous studies have indicated that the total saponin content in bitter gourd gradually declines during fruit development. However, the specific compositional profiles and the dynamic changes of individual triterpenoid saponins remain poorly understood (Qi et al., 2024). Furthermore, the underlying molecular mechanisms regulating the accumulation of these compounds throughout fruit development have yet to be fully elucidated.

The biosynthesis of triterpenoid saponins involves two primary stages: the formation of the triterpenoid scaffold and its subsequent structural modification. Initially, isopentenyl pyrophosphate (IPP) and dimethylallyl pyrophosphate (DMAPP), which are derived from the mevalonate (MVA) pathway in the cytoplasm and the methylerythritol phosphate (MEP) pathway in the plastids, are catalyzed by a series of enzymes, including geranyl diphosphate synthase (GPS), farnesyl diphosphate synthase (FPS), squalene synthase (SS), and squalene epoxidase (SQE), to synthesize the triterpenoid precursor 2,3-oxidosqualene. Subsequently, 2,3-oxidosqualene undergoes cyclization by various oxidosqualene cyclases (OSCs) to generate distinct scaffolds such as β-amyrin and cucurbitadienol. Finally, these scaffolds are subjected to structural modifications, primarily through oxidation by cytochrome P450 monooxygenases (CYP450s) and glycosylation by UDP-glycosyltransferases (UGTs), resulting in the structural diversity of triterpenoid saponins (Yao et al., 2020).

The cucurbitadienol-based cucurbitacin pathway has been well characterized in cucumber, melon and watermelon. Comparative genomic analyses of these three cucurbits revealed a conserved *CYP87D20* among cucumber, melon and watermelon, as well as a cucumber-specific *CYP88L2* (Zhou et al., 2016). In cucumber, a cucurbitacin C biosynthetic module consisting of nine genes has been identified, and four of the pathway enzymes have been characterized. The expression of cucurbitacin C biosynthetic genes is controlled by two tissue-specific basic helix–loop–helix (*bHLH*) transcription factors, which regulate bitterness in the leaves (*Bl*, bitter leaf) and fruit (*Bt*, bitter fruit). Mutations within the promoter of *Bt* effectively remove fruit bitterness (Shang et al., 2014). In bitter gourd, Takase et al. (2019) used RNA-Seq data and gene co-expression analysis to identify three *CYP450* genes (*CYP81AQ19*, *CYP88L7* and *CYP88L8*) potentially involved in cucurbitacin biosynthesis. Functional characterization in yeast showed that co-expression of *CYP81AQ19* with cucurbitadienol synthase produced cucurbita-5,24-diene-3β,23α-diol, that *CYP88L7* is a multifunctional enzyme catalyzing both C19 hydroxylation and the formation of the C5–C19 ether bridge characteristic of bitter gourd cucurbitacins, and that *CYP88L8* catalyzes C7 hydroxylation. This study demonstrated that allylic hydroxylation is a key enzymatic transformation that triggers subsequent processes leading to structural diversification. However, research on genes associated with triterpenoid saponin biosynthesis in bitter gourd remains limited. Qi et al. (2024) identified 14 candidate genes from transcriptome data across different fruit developmental stages that exhibited expression patterns correlated with saponin accumulation. These candidates include genes involved in the triterpene backbone biosynthetic pathway, such as *FPS1*, *SS12*, *SQE1*, and *CPQ*, as well as those encoding backbone-modifying enzymes, specifically *CYP97A3*, *CYP71AN24*, *UGT94E5*, and *UGT73C6*. In a separate study, Zhang (2024a) characterized 12 candidate *UGT* genes potentially involved in saponin biosynthesis; however, subsequent functional assays failed to demonstrate catalytic activity for these candidates. Xue et al. (2025) used a comparative transcriptomic and metabolomic analysis to systematically explore the metabolic and molecular basis of quality traits such as flavor, color and nutrition in bitter gourd. They found that the varying expression of unigenes encoding *DTPS*, *TTPS* and *GGPPS* was associated with the terpenoid profiles of the cultivars. However, this study did not perform targeted metabolomic profiling of terpenoids, and the identified genes did not include key backbone-modifying genes. Therefore, the specific saponin components and their regulatory genes underlying triterpenoid saponin accumulation in bitter gourd remain to be systematically elucidated.

Recent advances in multi-omics strategies have significantly accelerated the discovery and functional characterization of plant natural product biosynthetic pathways (Wan et al., 2025). Integrative approaches combining transcriptomics, metabolomics, and co-expression network analysis enable the systematic identification of candidate genes involved in the biosynthesis of valuable metabolites. However, these methods also have inherent limitations: correlation-based predictions require experimental validation. To date, several genome assemblies of bitter gourd have been published. The first genome of the cultivar ‘OHB3-1’ (ASM199503v1) was reported by Urasaki et al. (2017). Subsequently, the first T2T genome of the ‘Mca’ cultivar was reported (Fu et al., 2022), and most recently, a telomere-to-telomere (T2T) gap-free genome of the cultivar ‘Y52’ has been completed (Zhang et al., 2026). These genomic resources have greatly facilitated the identification of genes involved in secondary metabolite biosynthesis. In this study, we employed an integrated metabolomic and transcriptomic approach to characterize the shifts in the bitter gourd cultivar ‘RY45’ between 10 and 15 DPA. We investigated the differential expression of genes potentially involved in triterpenoid saponin accumulation. Furthermore, we conducted an exploratory co-expression network analysis to identify candidate transcription factors and structural genes, specifically focusing on *CYP450s* and *UGTs*, that may act as potential regulators of saponin accumulation. Notably, this study links specific saponin components with candidate biosynthetic genes and transcriptional regulators during bitter gourd fruit development, thereby expanding the current understanding of triterpenoid saponin biosynthesis in Cucurbitaceae and offering valuable insights for future metabolic engineering and quality improvement of this crop.

## Materials and methods

### Plant materials

The bitter gourd cultivar ‘RY45’ used in this study is a hybrid derived from the cross of BAL-22-31 (♀) and Shan-Ku-Gua-45 (♂), which was developed by our laboratory in previous studies (Wu et al., 2013) (**Supplementary Figure S1**). This cultivar was officially registered in Fujian Province, China, in 2013 (Registration No.: Minrencai 2013016), and its genetic resources are preserved at the National Germplasm Repository of Min-Tai Characteristic Crops (Zhangzhou) under accession number MTKY0016, ensuring the long-term availability of this genetic material. A voucher specimen (Deposition No. 2026-*M. charantia* - 350603-02) was deposited in the Traditional Chinese Medicine Resource Herbarium, College of Pharmacy, Fujian University of Traditional Chinese Medicine (Herbarium Code: FJTCM). The specimen was collected from the experimental field of the Institute of Subtropical Agriculture, Fujian Academy of Agricultural Sciences, Zhangzhou, Fujian, China, by Fan Junmiao on July 21, 2026. To better capture the dynamics of saponin accumulation, fruits were harvested at 5, 10, and 15 DPA, representing three distinct developmental stages. For each time point, six fruits were pooled as one biological replicate, and three biological replicates were collected. Following harvest, each fruit was halved: one portion was used for total saponin quantification, while the other was immediately snap-frozen in liquid nitrogen and stored at –80 °C for subsequent molecular analyses.

### Determination of total saponin content

A 1.0 g sample of dried bitter gourd powder was accurately weighed and transferred into a 50 mL centrifuge tube. The sample was extracted with 10 mL of 80% (v/v) ethanol using ultrasonication for 30 min. Following centrifugation at 12,000 rpm for 10 min, the supernatant was collected and diluted 20-fold with 80% ethanol. The solvent was removed via vacuum rotary evaporation. The resulting residue was reacted with 0.2 mL of freshly prepared 5% (w/v) vanillin-acetic acid and 0.8 mL of perchloric acid. The mixture was vortexed, incubated in a 60 °C water bath for 15 min, and immediately cooled in an ice-water bath for 30 min. After the addition of 5 mL of acetic acid, the mixture was vortexed again. Following a 20-min equilibration at room temperature, the absorbance was measured at 545 nm.

The vanillin-perchloric acid colorimetric method is the most widely used and pharmacopoeia-recognized approach for total saponin quantification. The measurement wavelength of 545 nm was determined based on full-wavelength scanning of the saponin standard and representative fruit samples from different developmental stages (5, 10, and 15 DPA), which confirmed 545 nm as the maximum reliable and stable absorption wavelength for both the standard and the test samples (**Supplementary Figure S2**). Three parallel solutions were prepared for each sample to ensure reproducibility. It should be noted that this vanillin-perchloric acid colorimetric method measures the total content of saponins based on the colorimetric reaction of triterpenoid aglycones, but it cannot distinguish between different saponin subtypes or individual saponin monomers.

### Terpenoid extraction and analysis

Bitter gourd fruits were collected at 10 and 15 days after flowering to detect terpenoids. A 30 mg sample powder was weighed, and 1500 μL of 70% methanol pre-cooled at –20 °C was added. The mixture was vortexed six times, with each vortex lasting 30 seconds and a 30 minute interval between successive vortexing events. After centrifugation at 12,000 rpm for 3 minutes, the supernatant was collected. The sample was then filtered through a 0.22 μm microporous membrane and stored in a sample vial.

The samples were analyzed by ultra performance liquid chromatography-tandem mass spectrometry (UPLC-MS/MS) using an ExionLC™ AD ultra performance liquid chromatography equipped with an Agilent SB-C18 (1.8 μm, 2.1 mm × 100 mm) column. The UPLC conditions were as follows: the column temperature was held at 40 °C, and the flow rate was held constant at 0.35 mL min^−1^. A gradient using 0.1% v/v formic acid–water as Buffer A and 0.1% v/v formic acid–acetonitrile as Buffer B was run as follows: 5% Buffer B from 0 to 1 min, 5–95% Buffer B from 1 to 9 min, 95% Buffer B from 9 to 10 min, 95–5% Buffer B from 10 to 11.10 min, and 5% Buffer B until 14 min. The electrospray ionization (ESI) source temperature was set to 500 °C. The ion spray voltage (IS) was set to +5500 V (positive ion mode) and –4500 V (negative ion mode). The ion source gas I (GSI), gas II (GSII), and curtain gas (CUR) were set to 50, 60, and 25 psi, respectively. The triple quadrupole (QQQ) scan was performed in multiple reaction monitoring (MRM) mode, and the CAD (collision-activated dissociation) gas (nitrogen) was set to Medium. The declustering potential (DP) and collision energy (CE) for each MRM ion pair were individually optimized. Based on the metabolites eluted within each time period, a specific set of MRM ion pairs was monitored for each period. For metabolite identification, the following four criteria were applied: (1) accurate mass of the metabolite, (2) MS/MS fragmentation pattern, (3) isotopic distribution of MS/MS fragments, and (4) retention time (RT). The MS/MS spectra and retention times of the metabolites were matched one by one against those in the MetWare Database (MWDB), a self-built database containing high-quality MS/MS spectra. The tolerance parameters were set as follows: MS mass tolerance at 20 ppm, MS/MS mass tolerance at 20 ppm, and RT tolerance at 0.2 min. Quantification was performed using the MRM peak areas, and the relative content of each metabolite was derived after chromatographic peak alignment and correction across samples. Differentially accumulated metabolites (DAMs) were identified based on the following criteria: fold change ≥ 2 or ≤ 0.5, P ≤ 0.05, and variable importance in the projection (VIP) ≥ 1, with false discovery rate (FDR) < 0.1 used as a supplementary reference criterion. For statistical analysis involving large-scale multiple hypothesis testing, FDR correction was applied to control the expected proportion of false positives among all significant results. Given that this study focused specifically on triterpenoid saponins, all downstream analyses were based exclusively on this metabolite class.

### Transcriptome sequencing and analysis

Total RNA was extracted from bitter gourd fruits at two developmental stages (RY45–10 DPA and RY45–15 DPA). Sequencing was performed on the MGI platform. To obtain high-quality clean reads, raw reads were processed using fastp (version 0.23.2) (Chen et al., 2018) under the following criteria: (1) reads containing adapter sequences were removed; (2) paired reads were discarded when the proportion of N exceeded 10% of the total bases in either read; (3) paired reads were discarded when the proportion of low-quality bases (Q ≤ 20) exceeded 50% of the total bases in either read. Subsequently, the clean reads were mapped to the bitter gourd reference genome (NCBI Assembly accession: GCF_001995035.1) using HISAT2 (v2.2.1) (Kim et al., 2019). This reference genome was selected as it was the most comprehensive assembly available at the time of our data processing. The genome assembly and annotation files were downloaded from the NCBI Genome database at https://www.ncbi.nlm.nih.gov/datasets/genome/GCF_001995035.1/. Gene expression levels were quantified as fragments per kilobase of transcript per million mapped reads (FPKM). For statistical analysis involving large-scale multiple hypothesis testing, FDR correction was applied to control the expected proportion of false positives among all significant results. Differentially expressed genes (DEGs) were identified based on the following criteria: P-value (FDR) < 0.05 and |log2(fold change)| > 1. DEGs involved in triterpenoid saponin biosynthesis were identified by mapping transcriptome-wide DEGs to a reference database of known pathway genes from *Arabidopsis thaliana*, *Panax ginseng*, and *Medicago truncatula* (Kyoto Encyclopedia of Genes and Genomes (KEGG) and NCBI BLASTp, E-value ≤ 1e^-5^) (Kanehisa et al., 2025; Camacho et al., 2009), focusing on *FPS*, *SQS*, *SE*, *OSCs*, *CYP450s*, and *UGTs*. KEGG pathway classification was performed using the MetWare Cloud Platform (https://cloud.metware.cn).

### WGCNA analysis and correlation analysis

Transcripts with low expression (fragments per kilobase of transcript per million mapped reads [FPKM] < 1) across all samples were first filtered out using the varFilter function from the R package genefilter (v1.90.0). Weighted gene co-expression network analysis (WGCNA) was then performed on the retained transcripts (FPKM ≥ 1 in at least one sample at 10 or 15 DPA) using the WGCNA R package (version 1.73) (Langfelder and Horvath, 2008). To determine the optimal soft-thresholding power, we used the pickSoftThreshold function to evaluate a range of candidate powers by calculating the scale-free topology fit index (R²) and mean connectivity for each power value. A power of 18 was selected, as it provided a reasonable balance between scale-free topology fit (R² = 0.715) and network connectivity (**Supplementary Figure S3**), allowing the retention of biologically meaningful gene-gene connections. Co-expression modules were identified via dynamic tree cutting with a minimum module size of 50 and a merge cut height of 0.25, yielding 13 modules.

To identify potential regulatory relationships among transcription factors, candidate triterpenoid biosynthetic genes, and triterpenoid saponins, we performed Pearson correlation analysis with stringent filtering criteria: |r| > 0.9 and P < 0.01. The constructed network was visualized using the MetWare Cloud Platform (https://cloud.metware.cn).

### Fluorescence RT-qPCR assays

Total RNA was extracted using the SPARKeasy Plant RNA Rapid Extraction Kit (Sparkjade, Shandong, China) according to the manufacturer’s instructions. Total RNA (1 µg) was used as a template to generate cDNA using the EasyScript^®^ First-Strand cDNA Synthesis SuperMix (TransGen, Beijing, China). The reverse transcription reaction was performed in a 20 µL system containing 10 µL of Reaction Mix, 1 µL of EasyScript^®^ RT/RI Enzyme Mix, 1 µg of total RNA, 1 µL of Oligo (dT), and ddH_2_O to a final volume of 20 µL. The cDNA was then diluted 5-fold with ddH_2_O, and 1 µL of the diluted cDNA was used as the template for RT-qPCR. RT-qPCR was performed using SYBR qPCR Master Mix (Vazyme, China) in a 20 µL reaction system containing 10 µL of SYBR qPCR Master Mix, 0.5 µL of forward primer, 0.5 µL of reverse primer, 1 µL of cDNA, and 8 µL of ddH_2_O. The RT-qPCR reaction was incubated at 95 °C for 1 min, followed by 40 cycles of 95 °C for 5 s, 60 °C for 15 s, and 72 °C for 15 s, followed by melting curve detection from 65 °C to 95 °C. The relative expression level of each gene was calculated using the 2^-ΔΔCt^ method with *McHMG1/2* as the reference gene, which has been validated as a stable reference gene in bitter gourd across different tissues and developmental stages (Zheng et al., 2023). The primer sequences used for RT-qPCR are listed in **Supplementary Table S1**. The amplification efficiency of each primer pair is presented in **Supplementary Table S2**, and all efficiencies were within the acceptable range of 90-110%.

### Transient gene expression in bitter gourd fruit of *McCYP72A219*

The vectors of 1300-empty and 1300-*McCYP72A219*-OE were transformed into Agrobacterium tumefaciens strain GV3101psoup, respectively. A colony was picked into 1 mL YEP liquid medium containing rifampicin (50 mg/L) and kanamycin (50 mg/L) and incubated overnight with shaking. Then 100 μL bacterial solution was added into 50 mL YEP liquid medium containing kanamycin (50 mg/L) and Rifampicin (50 mg/L). The mixture was incubated overnight at 28 °C until the OD600 reached 0.5. After that, the culture was centrifuged at 4000 rpm for 10 minutes to remove the supernatant, and the pellet was resuspended in the injection buffer (MS + 2% sucrose + 200 μM Acetosyringone) to OD600 0.75. After 2–3 h of activation at room temperature.

Agrobacterium tumefaciens cells containing each plasmid were injected from the bottom of each fruit using a 1 mL syringe, with approximately 1 mL per fruit delivered through a single injection point until the entire fruit flesh was visibly infiltrated. Injected fruits were left on the plant throughout the experimental period until harvest at 3 days post-injection. For each treatment (1300-empty vector and 1300-McCYP72A219-OE), three independent biological replicates were conducted. For each biological replicate, five fruits were pooled and used for saponin content analysis and gene expression analysis. Fruits at 5 days post-anthesis (DPA) were selected for injection because our preliminary experiments showed that younger fruits (e.g., 3 DPA) turned yellow, failed to expand, and exhibited poor viability after injection, whereas larger fruits (e.g., 7–10 DPA) had already undergone significant expansion and could not be completely infiltrated with the Agrobacterium suspension; the 5 DPA stage represented the optimal balance between transformation efficiency and fruit viability. The sampling time point at 3 days post-injection was selected with reference to the time frames commonly used in transient transformation studies in cucurbitaceous crops (Chen et al., 2021; Lin and Luo, 2007). The empty vector control was included to exclude the effects of Agrobacterium infiltration and the vector backbone on saponin content and gene expression.

### Phylogenetic analysis

Multiple sequence alignments were performed using ClustalW (Thompson et al., 1994) implemented in MEGA 12 (Kumar et al., 2024) with a gap opening penalty of 10, a gap extension penalty of 0.1, and the Gonnet protein weight matrix. Phylogenetic trees were constructed using the maximum-likelihood (ML) method implemented in MEGA 12, with the JTT substitution model, partial deletion, and a site coverage cutoff of 70%. Node support was assessed with 1,000 bootstrap replicates. All phylogenetic trees presented in this study were constructed using the same pipeline. The resulting trees were visualized and edited using iTOL (Letunic and Bork, 2021).

## Results

### Metabolic profiling of triterpenoids during fruit development in bitter gourd

We first measured the total saponin contents in bitter gourd fruits at 5, 10, and 15 DPA. As shown in **Supplementary Figure S4**, the total saponin content at 5 DPA was slightly but significantly higher than that at 10 DPA. However, at 15 DPA, the saponin content decreased sharply, and the fruits at 15 DPA had reached commercial maturity. These results indicate that the 10–15 DPA period represents a critical transition phase for saponin accumulation (**Figures 1A, B**). Therefore, we selected 10 DPA and 15 DPA for subsequent transcriptomic and metabolomic analyses to identify key regulatory genes and metabolites involved in this transition.

**Figure 1:**
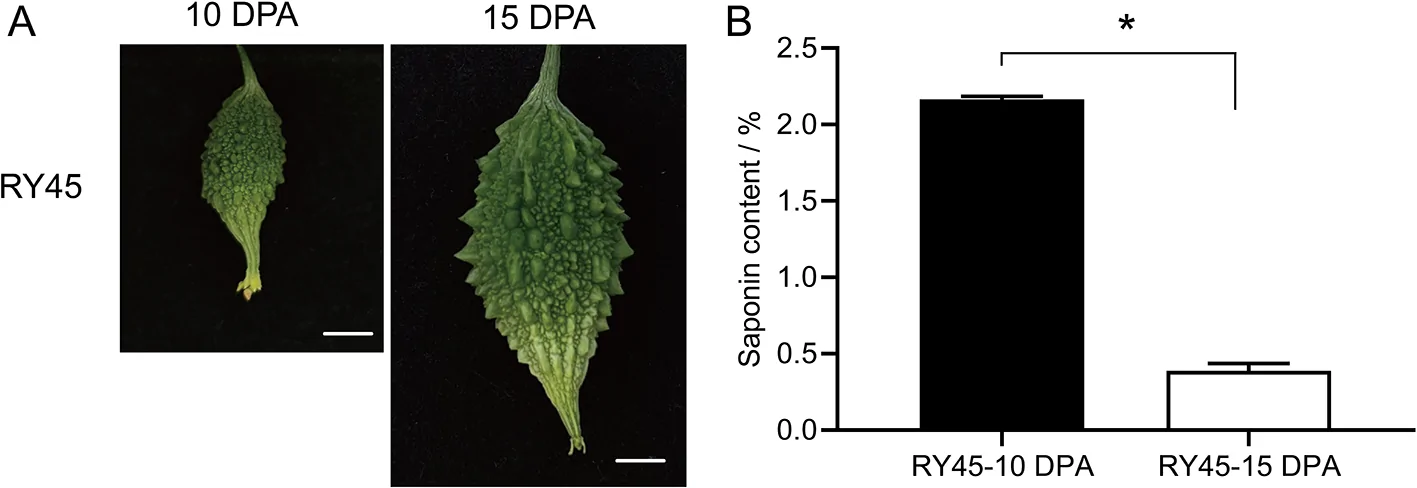
Changes in total saponin content in bitter gourd fruits. **(A)** Phenotypic appearance of bitter gourd fruits at 10 and 15 DPA. Bar, 1 cm. **(B)** Total saponin content in the fruits at 10 and 15 DPA. Data are shown as means ± SD (n = 3). Asterisks represent significant differences based on Student’s t-test: *P < 0.05; **P < 0.01.

Previous phytochemical studies have identified triterpenoid saponins as the essential bioactive compounds in bitter gourd (Jia et al., 2017). To investigate the profile and abundance of these compounds in ‘RY45’, we performed targeted terpenoid metabolomics analysis on fruit samples harvested at 10 and 15 DPA using UPLC-MS/MS. Principal component analysis (PCA) revealed that metabolic profiles were closely clustered according to developmental stage, indicating high experimental repeatability (**Figure 2A**). A comprehensive metabolomic analysis identified a total of 473 terpenoid metabolites in bitter gourd fruit. These metabolites were categorized into classes including sesquiterpenoids, monoterpenoids, diterpenoids, triterpenes, and triterpenoid saponins (**Figure 2B**). Following item-by-item verification against the published bitter gourd literature and confidence grading according to the Metabolomics Standards Initiative (MSI) (Sumner et al., 2007), 56 triterpenoid saponins were tentatively identified at MSI Level 2. These comprised cucurbitane-type (44.6%), oleanane-type (53.6%), and ursane-type (1.8%) (**Supplementary Table S3**). Saponins detected only at MSI Level 3 were excluded from the supplementary tables, as their annotations were limited to class-level confidence and certain skeletons lacked literature precedent in bitter gourd. Notably, the total content of triterpenoid saponins in ‘RY45’ fruits exhibited a significant decrease at 15 DPA compared with that at 10 DPA, with the cucurbitane-type saponins showing a particularly pronounced decline (**Figure 2C**).

**Figure 2:**
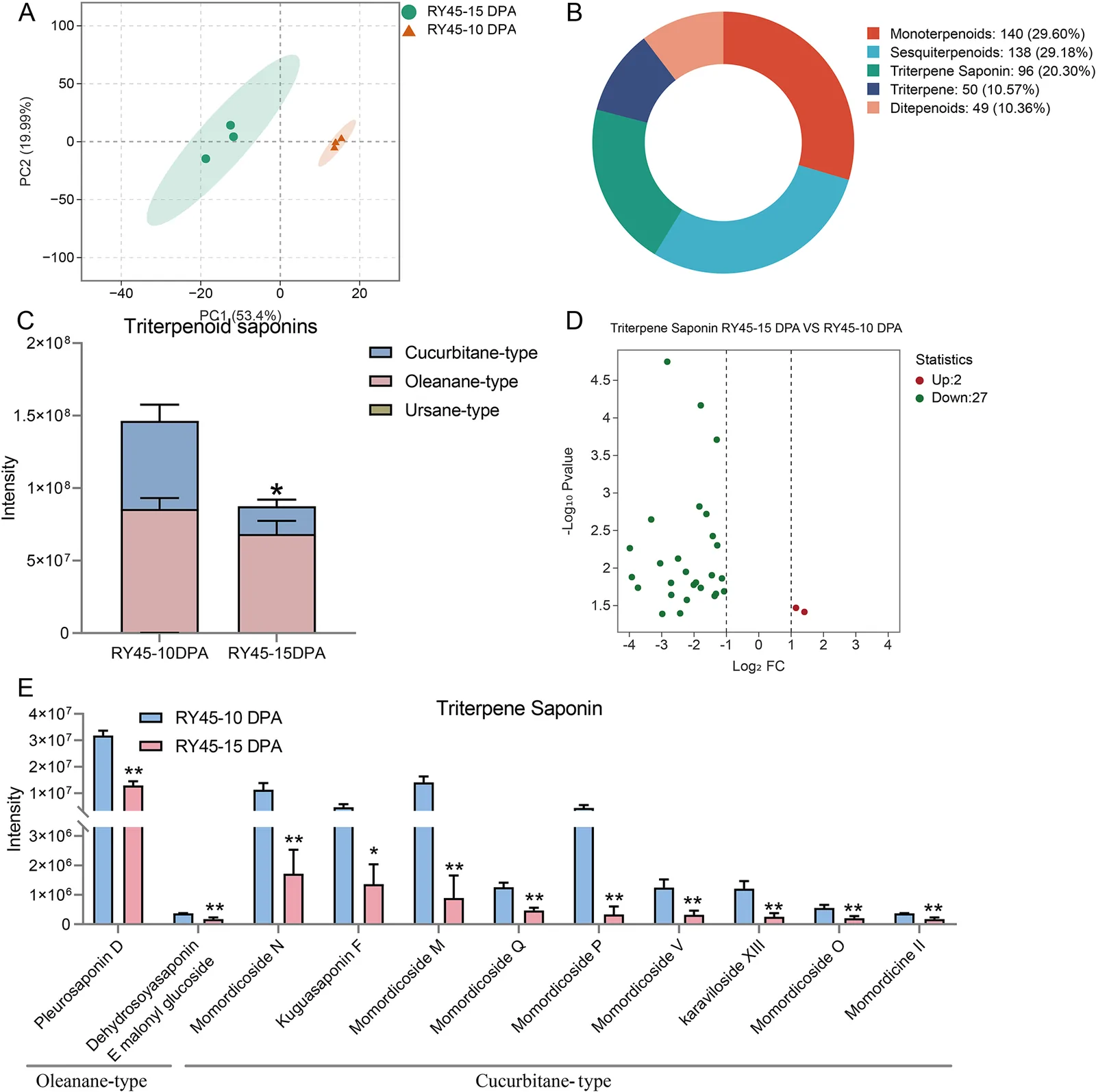
Metabolomic analysis of bitter gourd fruits at 10 and 15 DPA. **(A)** PCA of the metabolomic data. **(B)** Number of accumulated metabolites. **(C)** Compositional variations of triterpenoid saponins in bitter gourd. **(D)** Differentially accumulated triterpenoid saponins identified by comparing the metabolomic profiles of bitter gourd fruits at 10 and 15 DPA. **(E)** Content profiles of the triterpenoid saponin monomers at 10 and 15 DPA in bitter gourd fruits. Asterisks represent significant differences based on Student’s t-test: *P < 0.05; **P < 0.01.

To characterize the dynamic changes in the triterpenoid saponin profiles between RY45–10 DPA and RY45–15 DPA, we performed a comparative metabolomic analysis. DAMs were identified based on a fold change ≥ 2 or ≤ 0.5, P ≤ 0.05 and VIP ≥ 1. Metabolomic analysis revealed that 27 triterpenoid saponins were significantly downregulated, whereas only 2 were upregulated in the 15 DPA samples compared with those at 10 DPA (**Figure 2D**; **Supplementary Table S4**). To further control for false discovery rates in multiple testing, FDR correction was applied, with FDR < 0.1 used as a supplementary reference criterion for differential metabolite identification. The full FDR-adjusted results are available in **Supplementary Table S5**. Eleven triterpenoid saponins that were highly abundant, significantly decreased at 15 DPA, and identified with high-confidence spectral matching scores (MetWare score ≥ 0.7) were selected for subsequent analyses. These included cucurbitane-type saponins (momordicoside M, N, O, P, Q, V, momordicine II, karaviloside XIII, and kuguasaponin F) and oleanane-type triterpenoid saponins (tentatively identified as pleurosaponin D and dehydrosoyasaponin E malonyl glucoside, MSI Level 2) (**Figure 2E**; **Supplementary Figure S5**).

### Gene expression profiles in bitter gourd fruit during development

Gene expression patterns in bitter gourd fruits during development were profiled via RNA sequencing. A total of 334.11 million clean reads were obtained across the six samples. The clean reads were mapped to the bitter gourd reference genome (GCF_001995035.1), with total mapping rates ranging from 81.53% to 91.83%. Detailed information on clean read counts and mapping rates for each sample is provided in **Supplementary Table S6**. Comparative analysis between the 10 and 15 DPA stages identified 3,291 differentially expressed genes (DEGs) (P-value (FDR) < 0.05 and |log2(fold change)| > 1) (**Supplementary Table S7**), of which 1,643 were upregulated and 1,648 were downregulated (**Figure 3A**). Gene Ontology (GO) enrichment analysis revealed that these DEGs were significantly enriched in molecular functions, cellular components, and biological processes (**Figure 3B**). Notably, KEGG pathway analysis indicated significant enrichment in biosynthetic and metabolic pathways, with particular emphasis on terpenoid backbone biosynthesis, as well as sesquiterpenoid and triterpenoid biosynthesis (**Figure 3C**).

**Figure 3:**
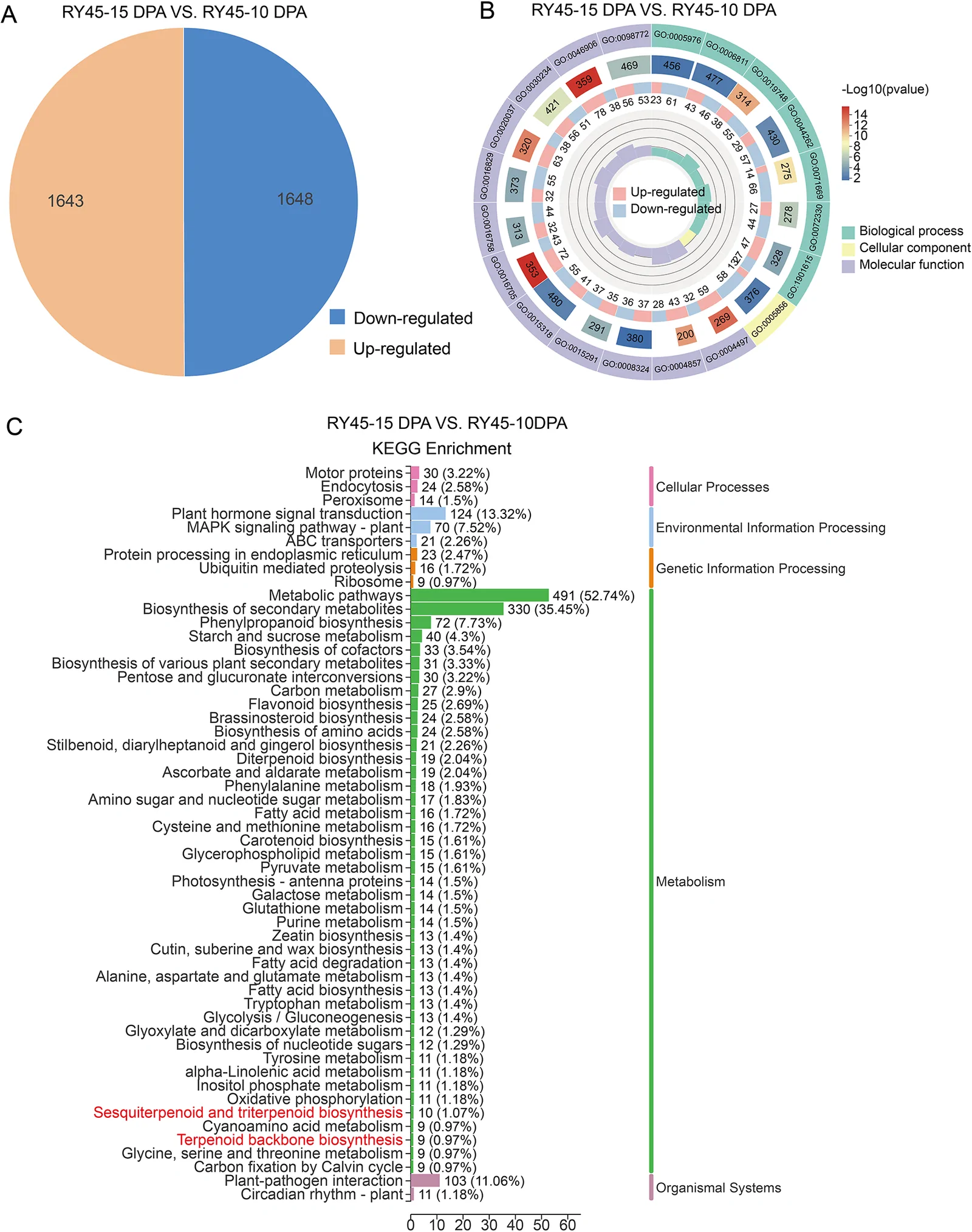
Transcriptomic analysis of bitter gourd fruits at 10 and 15 DPA. **(A)** The number of differentially expressed genes. **(B)** GO enrichment analysis of differentially expressed genes between 10 and 15 DPA. **(C)** KEGG pathway classification of differentially expressed genes between 10 and 15 DPA. The analysis was performed using the MetWare Cloud Platform (https://cloud.metware.cn). The bars represent the proportion of differentially expressed genes annotated to each KEGG pathway subcategory. Different colors represent different KEGG pathway categories.

### Candidate genes associated with triterpenoid saponin biosynthesis during bitter gourd fruit development

Given that the triterpenoid saponin content was significantly lower at RY45–15 DPA than at RY45–10 DPA, we investigated the molecular basis for these variations by analyzing the transcriptional profiles of genes potentially involved in triterpenoid saponin biosynthesis. KEGG pathway analysis revealed that the differentially expressed genes (DEGs) were significantly enriched in ‘sesquiterpenoid and triterpenoid biosynthesis’ as well as ‘terpenoid backbone biosynthesis’. A total of 50 DEGs were annotated as candidate genes associated with triterpenoid saponin biosynthetic pathways, including 1 *SQE* gene, 35 *CYP450* genes, 12 *UGT* genes, and 2 *OSC* genes (**Figure 4A**; **Supplementary Table S8**). Notably, several candidates exhibited high expression levels (FPKM > 10 in at least one sample) and were significantly downregulated, including three *CYP450s* (*McCYP86A7*, LOC111023447; *McCYP704C1*, LOC111016106; *McCYP72A219*, LOC111010820), two *UGTs* (*McUGT84B1*, LOC111017893; *McUGT73C6*, LOC111014244), and one *OSC* (*McβAS*, LOC111025306) (**Figure 4B**). Our RT-qPCR results further confirmed these RNA-seq findings (**Supplementary Figure S6**).

**Figure 4:**
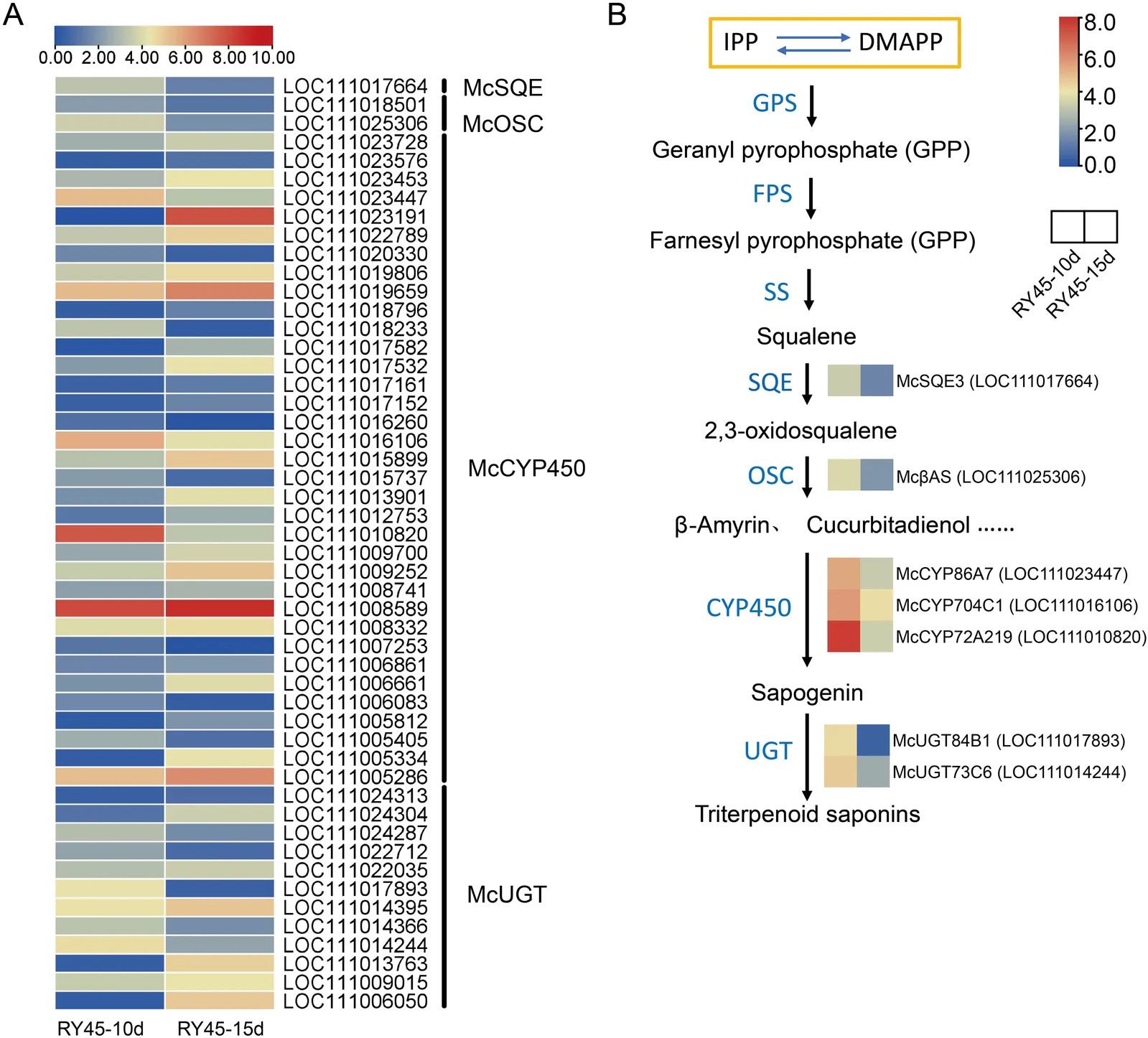
Transcriptional analysis of differentially expressed genes in the triterpenoid saponin biosynthetic pathway of bitter gourd fruit. **(A)** Heatmap showing the expression patterns of *McSQE*, *McCYP450*, *McUGT*, and *McOSC* genes in bitter gourd fruit at 10 and 15 DPA. The color scale represents Log_2_(FPKM) values, with blue indicating low expression and red indicating high expression. **(B)** Overview of the triterpenoid saponin biosynthetic pathway.

### Transcriptional regulation of bitter gourd fruit development

A comprehensive analysis of transcription factors during bitter gourd fruit development identified 247 differentially expressed genes (DEGs) belonging to 38 transcription factor families. Among these, 77 transcription factors across 21 families exhibited relatively high expression levels (FPKM > 10 at one or more developmental stages) (**Figure 5A**). Comparative analysis revealed that the expression of most transcription factors within the *AP2*, *bHLH*, *MYB*, *NAC*, *MADS*, and *WRKY* families was significantly downregulated at 15 DPA compared with 10 DPA. Conversely, the majority of transcription factors within the *bZIP*, *GRF*, and *B3* families showed a marked increase in expression levels at the 15 DPA (**Figure 5B**).

**Figure 5:**
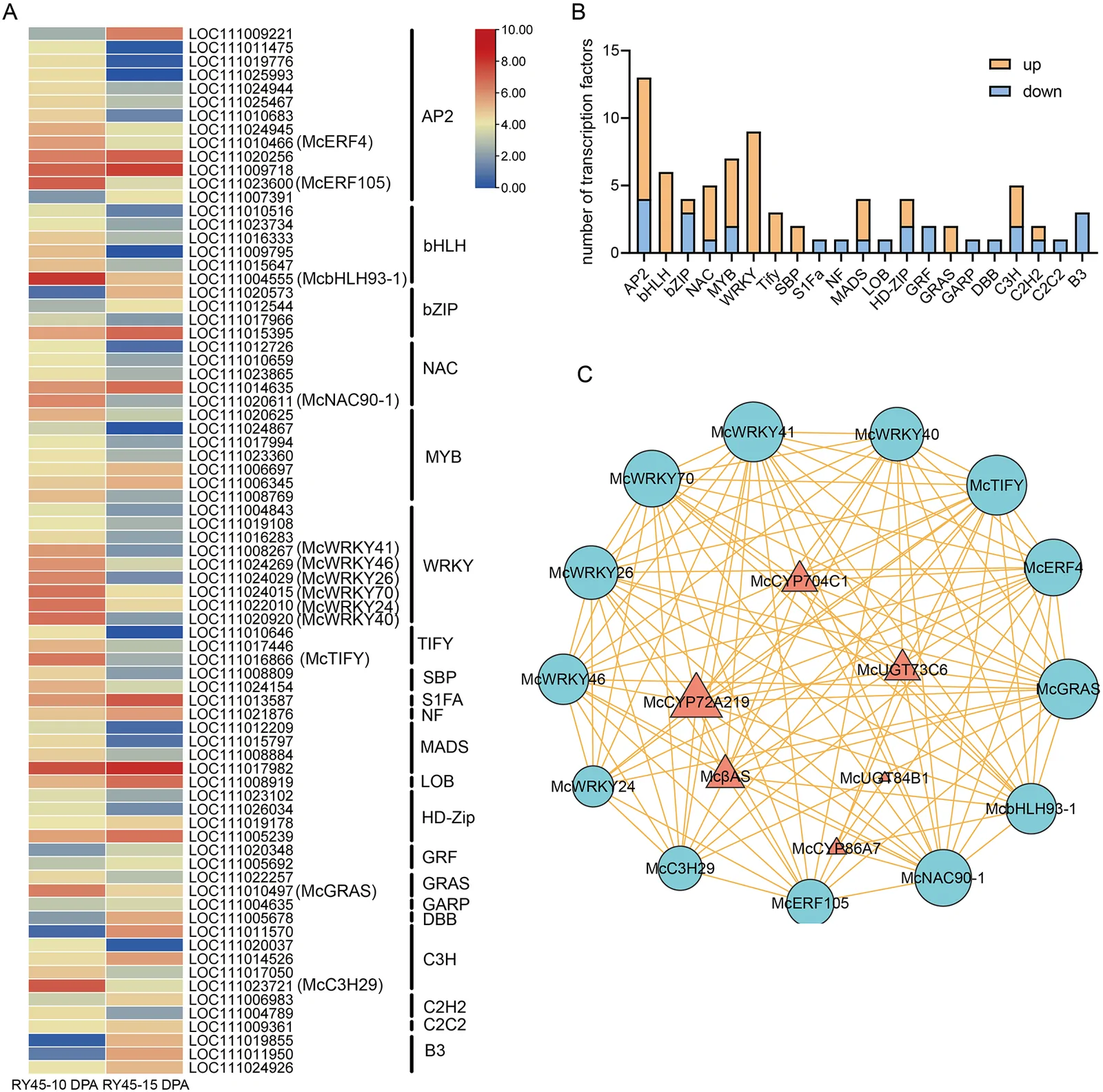
Transcriptional analysis of differentially expressed transcription factors in bitter gourd fruit. **(A)** Heatmap showing the expression profiles of identified transcription factors in fruit at 10 and 15 DPA. The color scale represents Log_2_(FPKM) values, with blue indicating low expression and red indicating high expression. **(B)** Statistics of up- and downregulated transcription factors across different families at 15 DPA compared to 10 DPA. **(C)** Co-expression network illustrating the regulatory relationships between transcription factors and genes involved in triterpenoid saponin biosynthesis. Red triangles represent triterpenoid saponin biosynthesis-related genes, blue circles represent transcription factors. Yellow lines indicate positive correlations.

Transcription factors are known to play a crucial role in regulating triterpenoid saponin biosynthesis, as previously reported (Jiang et al., 2024a). We performed an exploratory WGCNA analysis to identify potential transcriptional regulators of triterpenoid saponin biosynthesis-related genes. The results suggested that 36 candidate genes associated with triterpenoid saponin biosynthesis were clustered in the turquoise module (**Supplementary Figure S7**), among which 14 genes were significantly downregulated in bitter gourd fruits at 15 DPA, including *McCYP86A7*, *McCYP704C1*, *McCYP72A219*, *McUGT84B1*, *McUGT73C6*, and *McβAS*. Clustered together with these triterpenoid saponin biosynthetic-related genes were 13 transcription factors (each with FPKM > 50 in at least one sample) (**Supplementary Table S9**). These 13 transcription factors were classified into seven gene families, including *WRKY* (*McWRKY24*, LOC111022010; *McWRKY26*, LOC111024029; *McWRKY40*, LOC111020920; *McWRKY41*, LOC111008267; *McWRKY46*, LOC111024269; *McWRKY70*, LOC111024015), *AP2* (*McERF4*, LOC111010466; *McERF105*, LOC111023600), *TIFY* (*McTIFY*, LOC111016866), *NAC* (*McNAC90-1*, LOC111020611), *GRAS* (*McGRAS*, LOC111010497), *C3H* (*McC3H29*, LOC111023721), and *bHLH* (*McbHLH93-1*, LOC111004555). Correlation analysis of the 13 candidate transcription factors and the six downregulated DEGs related to the triterpenoid biosynthetic pathway (*McCYP86A7*, *McCYP704C1*, *McCYP72A219*, *McUGT84B1*, *McUGT73C6* and *McβAS*) revealed that all 13 transcription factors exhibited a significant positive correlation with *McCYP72A219*. Furthermore, no significant correlation was observed between *McUGT84B1* and the candidate transcription factors, while *McbHLH93–1* and *McNAC90–1* showed significant positive correlations with all five genes except *McUGT84B1* (**Figure 5C**).

### Correlation analysis of candidate triterpenoid biosynthetic genes and transcription factors associated with triterpenoid saponin accumulation

To identify potential regulatory relationships among candidate triterpenoid biosynthetic genes, transcription factors, and triterpenoid saponins, we performed Pearson correlation analysis. A total of 20 candidate triterpenoid biosynthetic genes (**Supplementary Table S10**), 14 transcription factors (**Supplementary Table S11**), and 11 triterpenoid saponins, which were highly abundant and significantly downregulated at 15 DPA, exhibited strong correlations (|r| > 0.9, P < 0.01). Among the biosynthetic genes, 11 showed a positive correlation with triterpenoid saponin content (**Figure 6A**). Notably, *McCYP72A219* exhibited the most significant downregulation at 15 DPA (**Figure 4B**) and was highly correlated with the accumulation of pleurosaponin D, momordicoside M, N, O, P, Q, V, momordicine II, karaviloside XIII, and dehydrosoyasaponin E malonyl glucoside. Correlation analysis revealed that 8 transcription factors (*McbHLH93-1*, *McNAC90-1*, *McWRKY26*, *McWRKY40*, *McWRKY41*, *McWRKY70*, *McERF4* and *McERF105*) were positively correlated with triterpenoid saponin content, whereas 6 transcription factors showed a negative correlation, suggesting that these transcription factors may be potentially involved in the regulation of triterpenoid saponin accumulation (**Figure 6B**). To preliminarily investigate the potential role of *McCYP72A219* in regulating the accumulation of bitter gourd saponins, we performed an Agrobacterium-mediated transient overexpression assay. At three days post-injection, the expression level of *McCYP72A219* in fruits injected with *McCYP72A219*-OE was increased more than nine-fold compared to the empty vector control (**Figure 7A**), and the total saponin content was also significantly elevated (**Figure 7B**). These results suggest that *McCYP72A219* may be associated with increased total saponin accumulation, although the specific saponin monomers affected remain to be determined. To further investigate the expression changes of other triterpenoid saponin biosynthesis-related structural genes as well as candidate transcription factors identified by WGCNA, we focused on the *bHLH*, *NAC*, *ERF*, and *WRKY* families that have been previously reported to participate in triterpenoid saponin biosynthesis in other plant species (Liu et al., 2023; Jiang et al., 2024b). RT-qPCR analysis revealed that, among all tested candidates, only *McERF105* was significantly upregulated in the *McCYP72A219* transient overexpression line (more than three-fold relative to the control), while the other tested transcription factors showed no significant expression changes (**Figure 7A**). Collectively, these findings suggest that transient overexpression of *McCYP72A219* is associated with increased total saponin accumulation and with the specific upregulation of *McERF105*.

**Figure 6:**
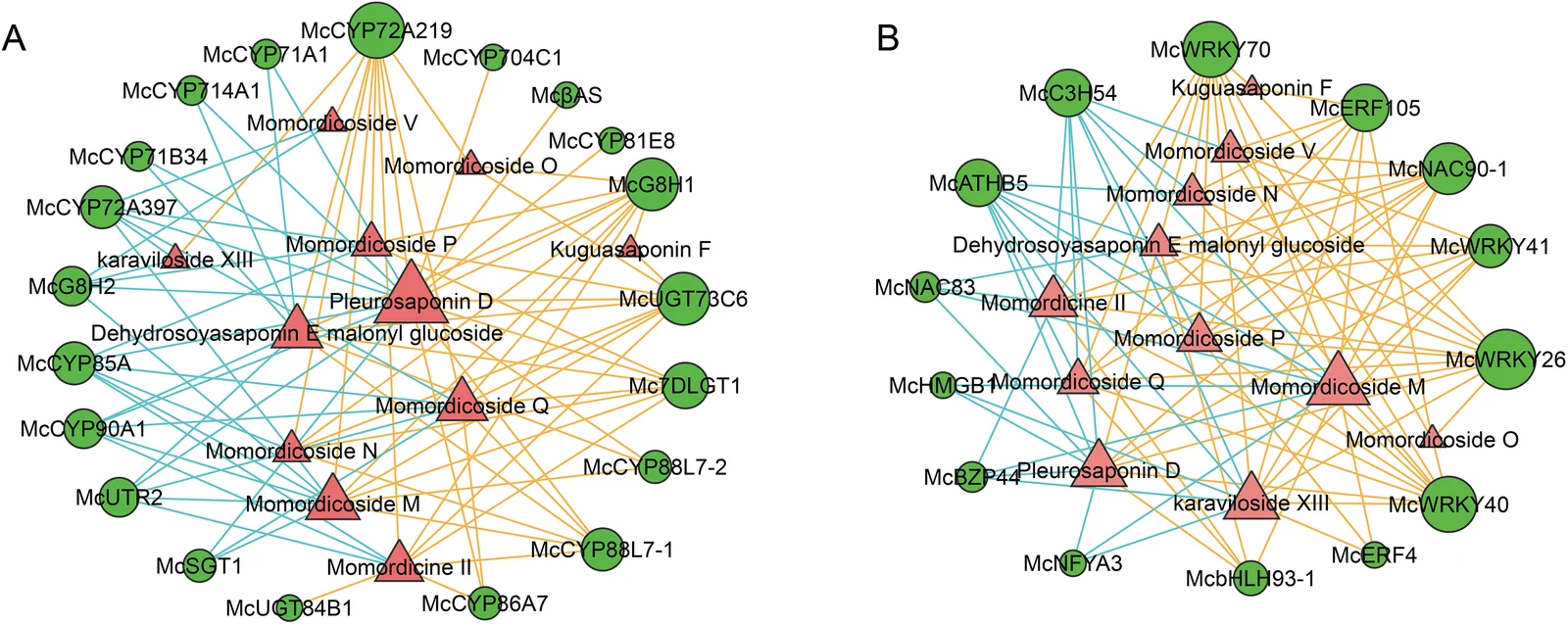
Correlation analysis between differentially expressed metabolites and genes in bitter gourd fruit. **(A)** Regulatory network of candidate structural genes and their association with triterpenoid saponin monomers. **(B)** Regulatory network of transcription factors associated with triterpenoid saponin monomers. Red triangles represent triterpenoid saponin and green circles represent triterpenoid saponin biosynthesis-related genes **(A)** and transcription factors **(B)**, respectively. Yellow and blue lines denote positive and negative correlations, respectively.

**Figure 7:**
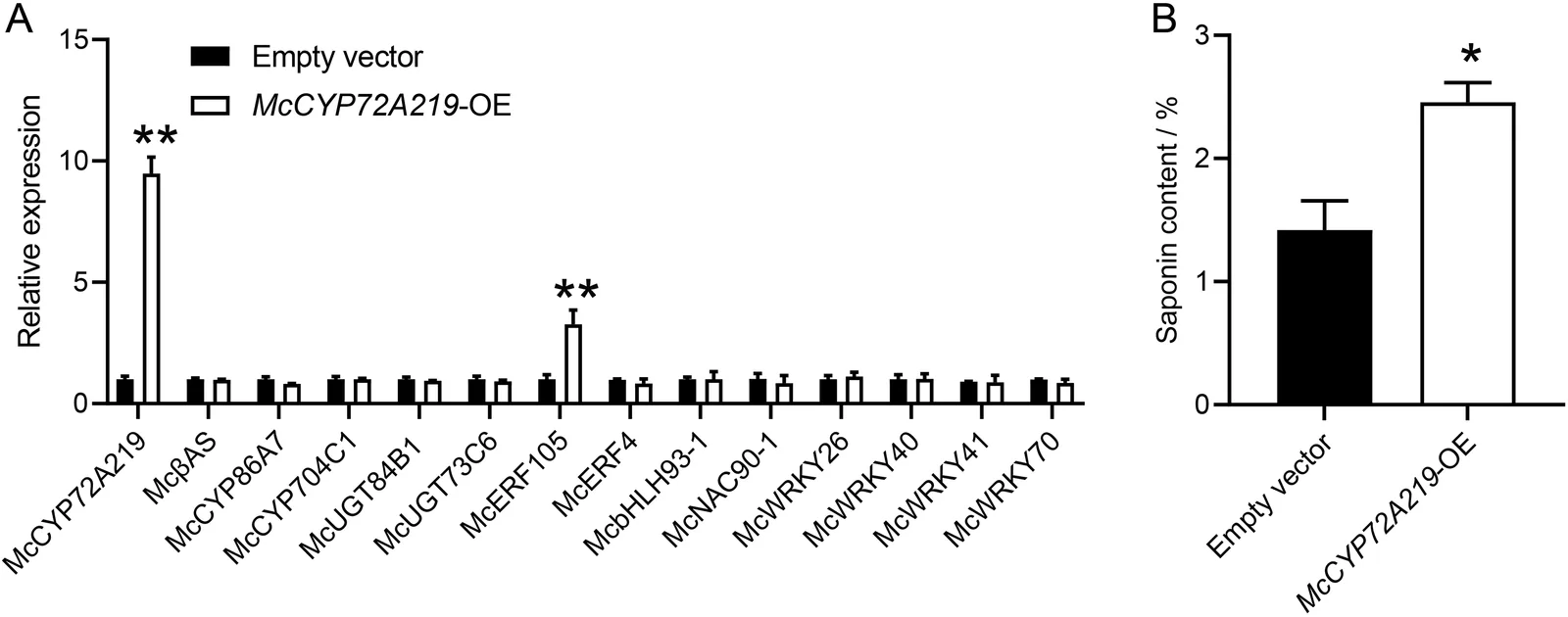
Transient overexpression of *McCYP72A219* in bitter gourd fruits. **(A)** Expression of *McCYP72A219* and other triterpenoid saponin biosynthesis-related genes in empty vector control and *McCYP72A219*-OE at 3 days after injection. **(B)** Saponin content in empty vector control and *McCYP72A219*-OE at 3 days after injection. Vertical bars represent the standard deviation of three biological replicates (five fruits pooled per replicate). Asterisks indicate significant differences based on Student’s t-test: *P < 0.05; **P < 0.01. OE, overexpression.

## Discussion

### Metabolic shift and triterpenoid saponin biosynthesis during bitter gourd fruit development

In this study, we employed an integrated multi-omics approach to elucidate the regulatory mechanisms underlying triterpenoid saponin accumulation in bitter gourd cv. ‘RY45’. Our targeted metabolomic profiling identified 473 terpenoid metabolites, including 56 triterpenoid saponins tentatively identified at MSI Level 2. Determination of total saponin content revealed a significant decline in bitter gourd fruits from 10 to 15 DPA, a finding that aligns with previous reports (Qi et al., 2024). From an ecological perspective, this decline may be associated with the plant’s “defense-dispersal trade-off” strategy. High concentrations of saponins in immature fruits protect seed development, whereas reduced content at the mature stage facilitates the attraction of frugivorous animals for seed dispersal (Whitehead et al., 2016). On this basis, further metabolic profiling demonstrated that cucurbitane-type saponins decreased significantly, whereas oleanane-type saponins as a class showed no significant change, although individual oleanane-type saponins (e.g., pleurosaponin D) decreased significantly. In the triterpenoid saponin biosynthetic pathway, different OSCs catalyze the conversion of 2,3-oxidosqualene into various triterpenoid precursors. Cucurbitane-type saponins are derived from cucurbitadienol, produced by cucurbitadienol synthase (CS). In our data, *CS* genes did not show significant changes (**Supplementary Table S12**). The significant downregulation of several cucurbitane-type saponins (momordicoside M, N, O, P, Q, V, momordicine II, kuguasaponin F, karaviloside XIII) in the absence of differential expression of *CS* genes presents an apparent disconnect. Cucurbitane-type saponin accumulation depends not only on backbone formation by *OSCs* but also on subsequent modifications catalyzed by *CYP450s* and *UGTs*. In our study, several *CYP450s* (*McCYP72A219*, *McCYP86A7*) and *UGTs* (*McUGT73C6*) were significantly downregulated at 15 DPA and showed strong positive correlations with cucurbitane-type saponin levels. Thus, the decline in cucurbitane-type saponins may be primarily driven by reduced activity of these downstream modifying enzymes rather than by the OSC step. This highlights that post-cyclization modifications can be rate-limiting for the accumulation of specific saponin types, even when the upstream cyclase genes are constitutively expressed. Furthermore, different members of the *CYP450s* and *UGTs* families may possess substrate preferences when participating in the modification of triterpenoid skeletons. For instance, *McCYP72A219* was positively correlated with multiple saponin monomers from both skeleton classes, including oleanane-type (pleurosaponin D, dehydrosoyasaponin E malonyl glucoside) and cucurbitane-type (momordicoside M, N, O, P, Q, V, momordicine II, karaviloside XIII) saponins, whereas *McCYP704C1* was significantly correlated only with the oleanane-type saponin pleurosaponin D (**Figure 6A**). Such differences in substrate specificity could also contribute to the observed divergence in accumulation profiles among saponin types.

### Candidate genes associated with triterpenoid saponin biosynthesis

Triterpenoid saponin biosynthesis comprises two distinct phases: the formation of the triterpene backbone and its subsequent modification. The triterpene backbone is catalyzed by GPS, FPS, SS and SQE, whereas the modification is primarily mediated by OSCs, CYP450s and UGTs. The *β-AS* gene, a member of the *OSC* family, encodes a key enzyme catalyzing the cyclization of 2,3-oxidosqualene into β-amyrin, which serves as a committed step in the biosynthesis of oleanane-type triterpenoids. The function of *β-AS* genes has been well-documented in various plant species, including *Panax ginseng*, *Glycyrrhiza uralensis*, *Arabidopsis thaliana*, *Panax japonicus*, and *Artemisia annua* (Yin et al., 2020; Liu et al., 2016). In the present study, we identified a homolog in bitter gourd, *McβAS*, and found that its expression level was significantly downregulated at 15 DPA compared to 10 DPA. Consistent with previous findings, our joint analysis of metabolomic and transcriptomic data showed a significant correlation between *McβAS* expression and the levels of the oleanane-type triterpenoid saponin, pleurosaponin D.

The *CYP716A* subfamily is closely associated with oleanane-type triterpene biosynthesis in various plants (Seki et al., 2015; Fukushima et al., 2011). Similarly, several *CYP72A* members have been functionally characterized in saponin biosynthesis. These include *MtCYP72A67* and *MtCYP72A68* from *Medicago truncatula*, which were confirmed *in vitro* and *in vivo* as hydroxylases/oxidases acting on the oleanane backbone (Fukushima et al., 2013; Biazzi et al., 2015); *CYP72A154* from *Glycyrrhiza uralensis*, which catalyzes three sequential C-30 oxidations of β-amyrin to produce glycyrrhetinic acid (Seki et al., 2011); *CYP72A63* from *Medicago truncatula*, also shown to catalyze C-30 oxidation of β-amyrin (Seki et al., 2011); and *CYP72A397* from *Kalopanax septemlobus*, which functions as an oleanolic acid 23-oxidase, converting oleanolic acid into hederagenin (Han et al., 2018). By contrast, seven *PgCYP72A* genes from *Platycodon grandiflorus* showed positive correlation with platycodin biosynthesis in transcriptome-metabolome profiling, but their enzymatic functions await experimental verification (Yu et al., 2025). In bitter gourd, Takase et al. (2019) functionally characterized *CYP81AQ19*, *CYP88L7* and *CYP88L8*, demonstrating their involvement in the oxidative modification of cucurbitacin aglycones. In the present study, we identified three distinct CYP450 candidates: *McCYP86A7*, *McCYP704C1*, and *McCYP72A219*. Specifically, *McCYP72A219* expression was positively correlated with pleurosaponin D, momordicoside M, N, O, P, Q, V, momordicine II, karaviloside XIII, and dehydrosoyasaponin E malonyl glucoside. Phylogenetic analysis placed *McCYP72A219* within the *CYP72* Clan, together with *MtCYP72A63*. Transient overexpression of *McCYP72A219* in bitter gourd fruits significantly increased total saponin content, although the specific saponin monomers affected remain to be determined. While CYP450s typically function as modifying enzymes expected to modify specific saponin aglycones rather than broadly elevate total saponins, the observed increase in total saponin content upon *McCYP72A219* overexpression has been documented in several plant species. For example, overexpression of *MtCYP72A68* in *Medicago truncatula* hairy roots significantly elevated total saponin levels (Tzin et al., 2019), and overexpression of *PgCYP716A264* in *Platycodon grandiflorus* hairy roots also increased total saponin content (Zhang et al., 2024b). These observations may reflect either enhanced direct catalytic efficiency or indirect effects on the entire metabolic pathway. To explore these possibilities, we examined the expression changes of triterpenoid saponin biosynthesis-related structural genes and candidate transcription factors in the overexpression fruits. Notably, the transcript levels of most structural genes showed no significant changes, whereas *McERF105* expression was upregulated more than three-fold. The regulatory role of AP2/ERF transcription factors in triterpenoid saponin biosynthesis has been demonstrated in other plant species. For instance, in *Panax ginseng*, Jiang et al. (2024b) confirmed that the *AP2/ERF* transcription factor *PgERF120* positively regulates ginsenoside biosynthesis. In Panax notoginseng, Deng et al. (2017) cloned and characterized *PnERF1*, showing that its overexpression upregulated the expression of triterpenoid saponin biosynthetic genes and significantly increased total saponin content. Notably, *McERF105* transcript levels significantly decreased from 10 DPA to 15 DPA and showed a significant positive correlation with the contents of multiple saponin monomers (**Figure 6B**). WGCNA analysis similarly demonstrated a positive correlation between *McCYP72A219* and *McERF105*. Collectively, these findings suggest a potential association between *McCYP72A219* and *McERF105* in saponin accumulation, although further functional verification is required to establish any causal relationship.

It should be acknowledged that the vanillin-perchloric acid colorimetric method used in this study measures total saponin content based on the colorimetric reaction of triterpenoid aglycones but cannot distinguish among different saponin subtypes or individual saponin monomers. Consequently, the precise catalytic activity, regio-selectivity, and direct target saponin monomers of *McCYP72A219* have not been fully elucidated. Owing to the lack of a well-established and reproducible stable genetic transformation system for bitter gourd, RNAi- or transient silencing-mediated loss-of-function validation was not performed in this study. In future work, we will actively explore feasible gene silencing systems for bitter gourd and perform targeted UPLC-MS/MS analyses of the monomeric saponin profile, combined with *in vitro* enzymatic assays, to decipher the precise catalytic function of *McCYP72A219* and validate its functional role in saponin biosynthesis.

The expression of *McCYP86A7* was positively correlated with the contents of pleurosaponin D, momordicine II, and momordicoside Q, and *McCYP704C1* expression showed a positive correlation with pleurosaponin D. Phylogenetic analysis revealed that both *McCYP86A7* and *McCYP704C1* belong to the *CYP86* Clan (**Supplementary Figure S8**). However, compared to the *CYP72* family, the functions of *CYP86* family members in triterpenoid saponin biosynthesis remain poorly understood. Whether the *CYP86* candidates identified here directly participate in saponin backbone modification or act through indirect regulatory effects warrants further investigation.

Since Achnine et al. (2005) first characterized the roles of *UGT73K1* and *UGT71G1* in triterpenoid saponin biosynthesis in *Medicago truncatula*, an increasing number of *UGT* family genes have been identified as participating in this pathway. Notably, members of the *UGT73* family primarily catalyze the glycosylation of oleanolic acid and hederagenin at the C-3 and C-28 positions (Augustin et al., 2012). In the present study, we identified a *UGT73* family gene in bitter gourd, designated *McUGT73C6*. We observed that the expression level of *McUGT73C6* was significantly downregulated in fruits at 15 DPA compared with those at 10 DPA, a trend consistent with the changes in the contents of pleurosaponin D, momordicoside M, momordicoside N, momordicoside O, momordicoside P, momordicoside Q, momordicine II, kuguasaponin F, and dehydrosoyasaponin E malonyl glucoside. Phylogenetic analysis revealed that both *McUGT73C6* and *Medicago truncatula* triterpenoid saponin biosynthesis gene *MtUGT73K1* are grouped into the *UGT* Group D (**Supplementary Figure S9**). Based on these observations, we postulate that *McUGT73C6* may be involved in triterpenoid saponin biosynthesis in bitter gourd. These correlations provide indirect evidence, and functional validation is required to establish causality.

In addition to reduced saponin biosynthesis, the significant decline in saponin content in bitter gourd fruits from 10 to 15 DPA may also be attributed to changes in saponin degradation. To explore this possibility, we examined the expression patterns of *β-glucosidase* (*BGLU*) genes, which encode key enzymes involved in saponin hydrolysis. Through KEGG annotation and homology-based screening, we identified 20 *BGLU* genes in our transcriptome (**Supplementary Table S13**). Among these, *McBGLU12* (LOC111021432) showed significant down-regulation, whereas *McBGLU46* (LOC111010739), *McBGLU25* (LOC111010372), and *McBGLU11* (LOC111013223) exhibited significant up-regulation from 10 to 15 DPA. Phylogenetic analysis revealed that *McBGLU46* clustered with the soybean saponin-hydrolyzing enzyme *GmSSBG1* (Feng et al., 2025), and *McBGLU12* grouped with the *Medicago truncatula* saponin-hydrolyzing enzyme *MtG1* (Lacchini et al., 2023), whereas *McBGLU11* clustered with the *Panax ginseng* saponin-hydrolyzing enzymes *PgGH1* and *PgGH2*, the *Panax notoginseng* saponin-hydrolyzing enzyme *PnGH1*, and the *Panax quinquefolium* saponin-hydrolyzing enzyme *PqGH1* (Ma et al., 2024) (**Supplementary Figure S10**). We further systematically screened the correlations between the expression levels of *McBGLU* family members and all MSI Level 2-identified triterpenoid saponin monomers that were significantly downregulated at 15 DPA (fold change ≥ 2, P ≤ 0.05, VIP ≥ 1). *McBGLU11* exhibited strong negative correlations with three saponin monomers: Kuguasaponin F (cucurbitane-type), Momordicoside O (cucurbitane-type), and Dehydrosoyasaponin E malonyl glucoside (oleanane-type) (**Supplementary Table S14**). *McBGLU25* showed a negative correlation specifically with Dehydrosoyasaponin E malonyl glucoside. *McBGLU11* emerged as the most compelling candidate degradation enzyme among the four *BGLU* members, based on both its phylogenetic affinity with known saponin-hydrolyzing enzymes and its significant negative correlations with multiple saponin monomers across two major skeleton types (cucurbitane-type and oleanane-type). It should be noted, however, that these correlations represent indirect evidence, and the direct hydrolytic activity and substrate specificity of *McBGLU11* require further validation through *in vitro* enzymatic assays.

The expression changes of *BGLU* genes suggest that the decline in saponin content in bitter gourd fruits at 15 DPA may be driven by a bidirectional regulatory mechanism: on one hand, the significant downregulation of biosynthetic genes limits saponin synthesis; on the other hand, the upregulation of candidate degradation genes such as *McBGLU11* may accelerate saponin hydrolysis. Notably, transient overexpression of *McCYP72A219* significantly downregulated the expression of both *McBGLU11* and *McBGLU46* (**Supplementary Figure S11**). Given that *McBGLU11* and *McBGLU46* are phylogenetically related to known saponin-hydrolyzing enzymes and *McBGLU11* expression showed strong negative correlations with multiple saponin monomers, this result raises the possibility that the elevation of total saponin content upon *McCYP72A219* overexpression may involve not only enhanced biosynthesis but also suppression of potential catabolic activities.

### Candidate transcription factors and their regulatory networks in triterpenoid saponin biosynthesis

Transcription factors serve as pivotal regulators in plant secondary metabolism. For instance, overexpression of *PgNAC41–2* and *PgERF120* in *Panax ginseng* has been shown to significantly enhance ginsenoside accumulation (Liu et al., 2023; Jiang et al., 2024b). In *Panax notoginseng*, *PnMYB1* and *PnMYB4* function as an activator and a repressor of ginsenoside biosynthesis, respectively. Specifically, *PnMYB4* antagonizes the transcriptional activation of *PnMYB1* by competitively binding to PnbHLH proteins. This process subsequently inhibits the expression of key biosynthetic genes (*PnSS*, *PnSE*, and *PnDS*) thereby reducing the accumulation of ginsenosides (Man et al., 2023). Our co-expression network analysis indicated that *McbHLH93–1* and *McNAC90–1* were positively correlated with the expression of *McβAS*, *McCYP86A7*, *McCYP704C1*, *McCYP72A219*, and *McUGT73C6. McERF105* was positively correlated with the expression of *McβAS* and *McCYP72A219*. The expression levels of *McbHLH93-1*, *McNAC90–1* and *McERF105* were downregulated in bitter gourd fruits at 15 days post-anthesis (DPA). Notably, the identification of putative bHLH-binding G-box motifs in the promoter regions of *McCYP86A7*, *McCYP704C1*, *McCYP72A219*, and *McUGT73C6* offers a hypothetical basis for a potential role of *McbHLH93–1* in triterpenoid biosynthesis, but this remains to be experimentally tested (**Supplementary Table S15**). Phylogenetic analysis showed that *McbHLH93–1* and *BpbHLH9* were classified into the same clade (**Supplementary Figure S12**). In *BpbHLH9* transgenic birch, the relative expression of genes encoding enzymes critical for triterpenoid synthesis showed different levels of up-regulation compared with wild-type birch (control). Specifically, the contents of betulinic acid, oleanolic acid, and betulin in *bHLH9–8* transgenic birch were increased by 11.35%, 88.34%, and 23.02%, respectively (Yin et al., 2017), compared to those in wild-type birch. Based on this correlative evidence and functional clues from homologs, we designate *McbHLH93–1* as a strong candidate positive regulator. However, these findings are correlative and do not directly demonstrate that *McbHLH93–1* regulates saponin biosynthesis in bitter gourd. We therefore emphasize that all proposed regulatory relationships presented in this study are correlative and hypothesis-generating; they do not constitute evidence of direct transcriptional regulation. To validate the direct regulatory functions of these candidate transcription factors, future studies could employ yeast one-hybrid assays, electrophoretic mobility shift assays, and dual-luciferase reporter assays to examine protein–DNA interactions, as well as generate stable transgenic overexpression or CRISPR-edited lines in a stable bitter gourd transformation system to assess the effects of these transcription factors on saponin accumulation. Furthermore, integrated transcriptomic and metabolomic analysis identified 14 transcription factors that are highly correlated with the accumulation of major saponins. These findings are consistent with a complex transcriptional regulatory framework in which these transcription factors may be involved in the coordinated activation or repression of triterpenoid saponin biosynthetic genes, potentially influencing the metabolic flux of the saponin biosynthetic pathway during fruit development. It should be noted, however, that the WGCNA analysis in this study was based on a relatively small sample size, and therefore all WGCNA-derived results should be interpreted as exploratory findings rather than definitive conclusions. Nevertheless, the two selected developmental stages represent a critical transition phase for saponin accumulation, as confirmed by prior phytochemical screening (5, 10, and 15 DPA). To minimize potential bias, a conservative soft-thresholding power (β = 18) was applied in the network construction, and the candidate genes identified by WGCNA were further cross-validated by transcriptome–metabolome correlation analysis. This convergence of independent analytical approaches supports the biological relevance of our WGCNA results. Nonetheless, we acknowledge that future studies with expanded sample sizes will be valuable to further consolidate the co-expression network presented here.

## Data Availability

The raw RNA-seq data have been deposited in the NCBI Sequence Read Archive (SRA) under BioProject accession number PRJNA1459662. BioSample accession numbers are SAMN57561245 (RY45-10 d-1), SAMN57561246 (RY45-10 d-2), SAMN57561247 (RY45-10 d-3), SAMN57561248 (RY45-15 d-1), SAMN57561249 (RY45-15 d-2), and SAMN57561250 (RY45-15 d-3). The metabolomics data generated in this study have been deposited in the MetaboLights database under accession number MTBLS15094 with sample names RY45-10 d-1, RY45-10 d-2, RY45-10 d-3, RY45-15 d-1, RY45-15 d-2, and RY45-15 d-3, and can be accessed via https://www.ebi.ac.uk/metabolights/MTBLS15094. The genome assembly and annotation files were downloaded from the NCBI Genome database (GCF_001995035.1; https://www.ncbi.nlm.nih.gov/datasets/genome/GCF_001995035.1/). The gene loci analyzed in this study are available in the NCBI Gene database under the following accession numbers: LOC111023447 (*McCYP86A7*), LOC111016106 (*McCYP704C1*), LOC111010820 (*McCYP72A219*), LOC111017893 (*McUGT84B1*), LOC111014244 (*McUGT73C6*), LOC111025306 (*McβAS*), LOC111022010 (*McWRKY24*), LOC111024029 (*McWRKY26*), LOC111020920 (*McWRKY40*), LOC111008267 (*McWRKY41*), LOC111024269 (*McWRKY46*), LOC111024015 (*McWRKY70*), LOC111010466 (*McERF4*), LOC111023600 (*McERF105*), LOC111016866 (*McTIFY*), LOC111020611 (*McNAC90-1*), LOC111010497 (*McGRAS*), LOC111023721 (*McC3H29*), LOC111004555 (*McbHLH93-1*), LOC111021432 (*McBGLU12*), LOC111010739 (*McBGLU46*), LOC111010372 (*McBGLU25*), and LOC111013223 (*McBGLU11*).
