# Supplementary material for: Integrating metabolomic and transcriptomic analyses to identify candidate genes and transcriptional regulators associated with triterpenoid saponin accumulation in developing Momordica charantia fruits
Source: Front Plant Sci. 2026 Oct 6;17:1868077. doi: 10.3389/fpls.2026.1868077 (PMC13682544; doi:10.3389/fpls.2026.1868077)
Supplement: Supplementary file 1 [file DataSheet1.docx]

Supplementary Material

**Supplementary Figure S1.** Overall morphology of the bitter gourd cultivar 'RY45'. (A) Whole plant; (B) Representative stems, leaves, flowers, and fruits.

**Supplementary Figure S2.** Full-wavelength scanning spectra of the saponin standard and total saponin extracts from bitter gourd fruits harvested at 5, 10, and 15 DPA.

**Supplementary Figure S3.** Determination of soft-thresholding power in the WGCNA. (A) The analysis of the scale-free fit index for various soft-thresholding powers. (B) The analysis of the mean connectivity for various soft-thresholding powers.

**Supplementary Figure S4.** Total triterpenoid saponin content in the fruits at 5, 10 and 15 DPA. Data are shown as means ± SD (n = 3). Different lowercase letters (a, b) denote significant differences at P < 0.05; different uppercase letters (A, B) denote highly significant differences at P < 0.01 (one-way ANOVA with Duncan's post-hoc test).

**Supplementary Figure S5.** MS/MS spectra of the triterpenoid saponins identified in bitter gourd fruits: (A) pleurosaponin D, (B) momordicoside M, (C) momordicoside N, (D) momordicoside O, (E) momordicoside P, (F) momordicoside Q, (G) momordicoside V, (H) momordicine II, (I) karaviloside XIII, (J) kuguasaponin F, and (K) dehydrosoyasaponin E malonyl glucoside.

**Supplementary Figure S6.** Expression of triterpenoid saponin biosynthesis-related genes in bitter gourd fruits at 10 and 15 DPA by RT-qPCR. Values are means ± SD of three independent biological replicates. Asterisks indicate significant differences (*p < 0.05 and **p < 0.01).

**Supplementary Figure S7.** WGCNA dendrogram and module assignment. The dendrogram shows hierarchical clustering of genes. The color bars below indicate 13 co-expression modules. The turquoise module contains 36 triterpenoid saponin-related genes.

**Supplementary Figure S8**. Phylogenetic tree of *CYP450s* from *Momordica charantia* (Mc), *Arabidopsis thaliana* (At), *Avena strigosa* (As), *Medicago truncatula* (Mt), *Glycine max* (Gm), *Glycyrrhiza uralensis* (Gu), *Panax ginseng* (Pg), *Lotus japonicus* (Lj), *Artemisia annua* (Aa), *Cucumis sativus* (Cs), *Arachis hypogaea* (Ah). The tree was constructed using the maximum likelihood method with 1,000 bootstrap replicates. Bootstrap values (≥ 0.6) are shown at each node. The tree is presented as a cladogram with equalized branch lengths, primarily illustrating topological clustering relationships rather than evolutionary distances. Accordingly, a scale bar indicating genetic distance is not applicable.

**Supplementary Figure S9.** Phylogenetic tree of *UGTs* from *Momordica charantia* (Mc), *Arabidopsis thaliana* (At), *Panax ginseng* (Pg), *Medicago truncatula* (Mt), *Glycine max* (Gm), *Glycyrrhiza uralensis* (Gu), *Barbarea vulgaris* (Bv). The tree was constructed using the maximum likelihood method with 1,000 bootstrap replicates. Bootstrap values (≥ 0.6) are shown at each node. The tree is presented as a cladogram with equalized branch lengths, primarily illustrating topological clustering relationships rather than evolutionary distances. Accordingly, a scale bar indicating genetic distance is not applicable.

**Supplementary Figure S10.** (A) Heatmap showing the expression profiles of *Momordica charantia BGLU* genes in fruit at 10 and 15 DPA. The color scale represents Log₂(FPKM) values, with blue indicating low expression and red indicating high expression. (B) Phylogenetic tree of *BGLUs* from *Momordica charantia* (Mc), *Glycine max* (Gm), *Panax ginseng* (Pg), *Medicago truncatula* (Mt), *Panax notoginseng (Pn), Panax quinquefolius* (Pq). The tree was constructed using the maximum likelihood method with 1,000 bootstrap replicates. Bootstrap values (≥ 0.6) are shown at each node. The tree is presented as a cladogram with equalized branch lengths, primarily illustrating topological clustering relationships rather than evolutionary distances. Accordingly, a scale bar indicating genetic distance is not applicable.

**Supplementary Figure S11.** Expression of *BGLU* genes in empty vector control and *McCYP72A219*-OE at 3 days after injection. Vertical bars represent the standard deviation of three biological replicates (five fruits pooled per replicate). Asterisks indicate significant differences based on Student's t-test: *, P < 0.05; **, P < 0.01. OE: overexpression.

**Supplementary Figure S12.** Phylogenetic tree of *bHLHs* from *Momordica charantia* (Mc), *Panax ginseng* (Pg), *Glycyrrhiza uralensis* (Gu), *Betula platyphylla* (Bp). The tree was constructed using the maximum likelihood method with 1,000 bootstrap replicates. Bootstrap values (≥ 0.6) are shown at each node. The tree is presented as a cladogram with equalized branch lengths, primarily illustrating topological clustering relationships rather than evolutionary distances. Accordingly, a scale bar indicating genetic distance is not applicable.
